# Identification of a novel *Ungulate copiparvovirus 10* in sheep of Hami, East Xinjiang, China

**DOI:** 10.3389/fvets.2026.1678726

**Published:** 2026-02-24

**Authors:** Juanjuan Pan, Haichun Jiang, Shengjin Luo, Lei Zhang, Guiling Wu, Wen Li, Shudong Cai, Yang Mei, Xintong Chen, Baoyu Chen, Weiwei Zhang, Panpan Tong, Jinxin Xie

**Affiliations:** 1 Laboratory of Animal Etiology and Epidemiology, College of Veterinary Medicine, Xinjiang Agricultural University, Urumqi, China; 2 Dahe Town Agricultural (Livestock) Development Service Center, Barkun County, Hami, China; 3 Animal Husbandry Station of Hami City, Hami, China; 4 Aksu Regional Animal Disease Control and Diagnostic Center, Aksu, China; 5 Yiwu County Livestock Work Service Center, Hami, China; 6 Guangzhou Kexiang Biological Technology Co., Ltd, Guangzhou, China; 7 Tacheng Vocational and Technical College, Tacheng, China; 8 Xinjiang Key Laboratory of New Drug Research and Development for Herbivores, Urumqi, China; 9 Key Laboratory for Animal Disease Detection, College of Animal Sciences, Yili Vocational and Technical College, Yili, China

**Keywords:** China, East Xinjiang, novel, sheep, *Ungulate copiparvovirus 10*

## Abstract

*Parvovirinae* viruses are a subfamily of the *Parvoviridae* family that can infect various vertebrate hosts and cause infections ranging from asymptomatic to severe disease. This study performed a metagenomic assessment of the sheep sera virome to evaluate emerging and exotic viruses in border zones, and identified a novel copiparvovirus. The DNA of Ovine copiparvovirus (OVPV) was only observed in the serum of sheep in Dahe Town of Hami City. Furthermore, the region-dependent prevalence was 10.4% (96/807) from 2022 to 2024. The OVPV genome was 5,219 nucleotides (nt) long and shared 99.3% nt identity with two bovine parvovirus 2 SXO335parvoV2 and SXO338parvoV (GenBank accession numbers: MZ244302 and MZ244302), reported in the ticks collected from China. Comparison of NS1 protein showed that two OVPVs obtained in this study had 99.8%–99.9% amino acid homology with the tick-derived bovine parvoviruses, which had not been classified within the genus *Copiparvovirus* and then provisionally designated “*Ungulate copiparvovirus 10*,” because they are far distant from other 10 species in *Copiparvovirus* genus with 48.5%–72.8% homology identified. Phylogenetic analysis further confirmed the classification of the OVPVs as a new species in the genus *Copiparvovirus*.

## Introduction

The *Parvoviridae* family includes non-enveloped, small single-stranded DNA (ssDNA) viruses with about 5–6 kb genomes (1, 2). Their genomes comprise two primary expression cassettes, with open reading frames (ORFs) on the left yielding non-structural (NS) proteins, while mRNAs that translate structural proteins (VPs) are transcribed from the right cassette (1, 2). As described in the guidelines of ICTV classification based on NS1, the family *Parvoviridae* includes three subfamilies: *Hamaparvovirinae*, *Densovirinae*, and *Parvovirinae*. The *Parvovirinae* and *Densovirinae* are distinguished primarily by their ability to infect vertebrate and invertebrate hosts, respectively, whereas the Hamaparvovirinae infect both vertebrates and invertebrates (1, 2).

The Parvovirinae subfamily is further subdivided into 11 genera (ICTV Taxonomy release 2018),^1^ including the *Copiparvovirus* genus (1, 2). Currently, nine species are recognized in the *Copiparvovirus* genus, including *Uungulate copiparvoviruses 1* (bovine parvovirus 2), *2* (porcine parvovirus 4), *3* (roe deer copiparvovirus), *4* (porcine parvovirus 6), *5* (copiparvovirus 101), *6* (equine parvovirus H), *7* (Eqcopivirus EqCoPV_8), and *8* (horse parvovirus CSF), as well as *Pinniped copiparvovirus 1* (Sesavirus) (2–13). Of these, the *Ungulate 2* and *4 copiparvoviruses* are responsible for circovirus-associated disease, abortions, and stillbirths in pigs. *Ungulate copiparvoviruses 6*, *7*, and *8* infect horses, resulting in hepatitis. *Ungulate copiparvovirus 5* causes sheep abortion, and the *Pinniped 1 copiparvovirus*, which infects sea lions (*Zalophus californianus*), leading to malnutrition and pneumonia. The *ungulate 1* and *3* viruses have been detected in ticks, cattle, and roe deer (2–13). Currently, in China, the *ungulate 1, 2, 4, 6, 7*, and *8 copiparvoviruses* have been identified in horses, cattle, pigs, and ticks (4, 9, 10).

This study comprehensively analyzed the occurrence of emerging viruses in ruminant livestock (cattle and sheep) in China–Mongolia border zones. The high-throughput sequencing (HTS) identified a new *Ungulate copiparvovirus 10* in sheep sera, which was spreading in Dahe Town, Hami City, Xinjiang, China. This is China’s first research study on ovine parvovirus (OVPV).

## Materials and methods

### Sample collection

Serum samples were collected from 2,068 healthy sheep and 1,780 healthy cattle from Hami City, Tacheng City, and Akesu City of Xinjiang, China, during 2022, 2023, and 2024. All the samples were stored at −80 °C until subsequent assessments (Table 1).

**Table 1:** Information of samples included in this study.

Location		Breed	Time	Sample number	Positive rate of OVPV	Cycle threshold values
Hami City	Dahe Town of Baliku County	Sheep	2022	312	13.78% (43/312)	8–22
			2023	204	3.92% (8/204)	18–20
			2024	291	15.46% (45/291)	18–20
		Cattle	2022	110	0%	>35
			2023	90	0%	
			2024	210	0%	
	Yiwu County	Sheep	2022	220	0%	
			2023	150	0%	
			2024	154	0%	
		Cattle	2022	105	0%	
			2023	260	0%	
			2024	305	0%	
Akesu City		Sheep	2022–2024	367	0%	
		Cattle	2022–2024	400	0%	
Tacheng City		Sheep	2022–2024	370	0%	
		Cattle	2022–2024	300	0%	
		Total		3,848	2.49%	

### Viral metagenomic analysis

For meta-transcriptomics (MTT), an RNA library was constructed. Briefly, 50 μL of serum from 106 samples was pooled and ultracentrifuged to collect the pellet, which was then resuspended in 200 μL of PBS. Then 100 μL of PK-15 cell lysate was treated with Trizol reagent (Tiangen, China) to increase the RNA concentration (>500 ng). The suspension was further mixed with 1,100 μL of Trizol reagent, and the total RNA was extracted. Subsequently, ribosomal RNA (rRNA) was removed using the Epicenter Ribo-zero rRNA Removal Kit (Epicenter, USA), followed by ethanol precipitation to remove rRNA-free residues. Then, sequencing libraries were generated with the rRNA-depleted RNA by following the protocol provided in the NEBNext Ultra Directional RNA Library Prep Kit (NEB, USA) (14).

For establishing a DNA library by multiple displacement amplification (MDA), DNA was extracted from the filtered supernatant using the DNeasy Blood & Tissue Kit (QIAGEN GmbH, Germany), and the sequencing library was constructed using the GenomiPhi V2 DNA Amplification Kit (Cytiva, UK). DNA concentrations were determined using the Qubit 1x dsDNA HS Assay Kit (Invitrogen, USA). The constructed RNA and DNA libraries were sequenced using the Illumina NovaSeq 6000 platform (Novogene, Tianjin, China), with 6G data obtained for each library; the sequencing depth is enough for screening the viruses (14).

Employing BWA 0.7.17, the cleaned data produced by HTS were first aligned with a swine genome sequence. The unaligned segments were removed using Samtools 1.17. The purified gene data were *de novo* assembled into contigs utilizing Megahit 1.2.9 with the Kmer iterative DBG approach, and subsequently aligned and annotated in the nr and nt libraries of NCBI using Diamond 2.1.8 and Nucleotide–Nucleotide BLAST 2.13.0+. The annotation findings were verified online using the Blastx module provided by the NCBI database. Following the screening and elimination of nonviral genetic sequences, the verified viral sequences were aligned with the obtained contigs to quantify the number of viral reads utilizing Bowtie2 version 2.4.5 (14).

### OVPV analysis

Total viral nucleic acid was extracted from each of the sera using the Viral RNA/DNA Kit (Geneaid, China). The presence of OVPV DNA in the samples was detected *via* TaqMan-MGB qPCR using primers and probes for the *NS1* gene (Supplementary Table S1). Subsequently, the quantitative polymerase chain reaction (qPCR) was performed using a 20 μL reaction mixture [containing 10 μL of 2 × SuperReal PreMix (TransGen Biotech), 0.1 μM primers and probe for OVPV (listed in Supplementary Table S1), and 1 μL of viral nucleic acids, made up to 20 μL with deionized water]. For target gene amplification, the parameters were as follows: initial denaturation for 15 min at 95 °C, followed by 45 cycles of denaturation for 15 s at 95 °C, annealing, and extension at 60 °C for 30 s.

### Amplification of the whole-genome sequence of OVPV

The whole-genome sequences of OVPVs were determined by PCR in samples positive for viral nucleic acids. Primers were designed utilizing the publicly accessible bovine parvovirus 2 reference strain SXO335parvoV2 sequences from GenBank (accession no.: MZ244301, Supplementary Table S1). The PCR settings and sequencing of positive amplicons were conducted as previously described (9). PCR reactions were carried out using a 50 μL reaction mixture comprising 25 μL of 2 × TransStart^®^ FastPfu Fly PCR SuperMix (TransGen Biotech), 0.5 μM of each forward and reverse primer specific to the individual viruses (listed in Supplementary Table S1), and 1 μL of viral nucleic acids, and deionized water to make the volume 50 μL. The PCR protocol consisted of an initial denaturation at 98 °C for 1 min, followed by 35 cycles of denaturation at 98 °C for 10 s, annealing at 50 °C for 5 s, and extension at 72 °C for 30 s, leading to a final extension at 72 °C for 1 min (TransGen Biotech).

### Isolation and screening of OVPV

For the *in vitro* analysis of OVPV, sheep kidney (SK) and lamb testis (LT) cells were used (15). Briefly, the qPCR-positive samples were centrifuged at 12,000×*g* for 3 min to collect the supernatants, which were filtered through a 0.22 μm filter and utilized to inoculate SK cells at 37 °C for 2 h in 5% CO_2_. Subsequently, the inoculum was replaced with DMEM augmented with fetal bovine serum (FBS; 2%) for 72 h. To harvest the viruses, the cells were subjected to three rounds of freeze and thaw, followed by repeated inoculation for six passages. Cytopathic effect (CPE) analysis was carried out daily after the inoculation.

### Phylogenetic analyses

**Figure 1:**
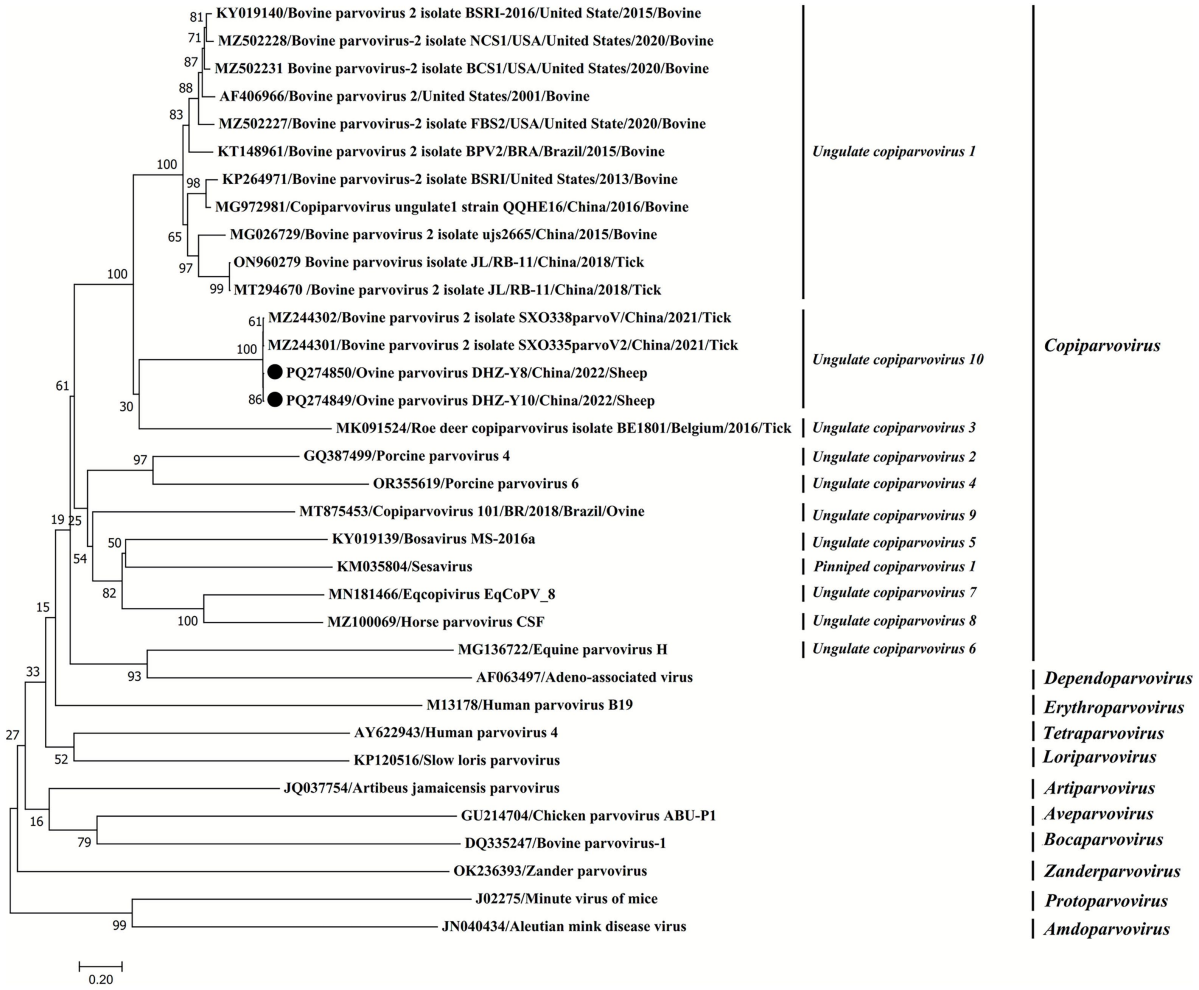
Phylogenetic tree based on the complete genomic sequences of parvoviruses. Phylogenetic analysis was carried out using the maximum-likelihood (ML) method using MEGA7 software (1,000 bootstrap replicates). Fixed circles indicate the viruses isolated in this study.

**Figure 2:**
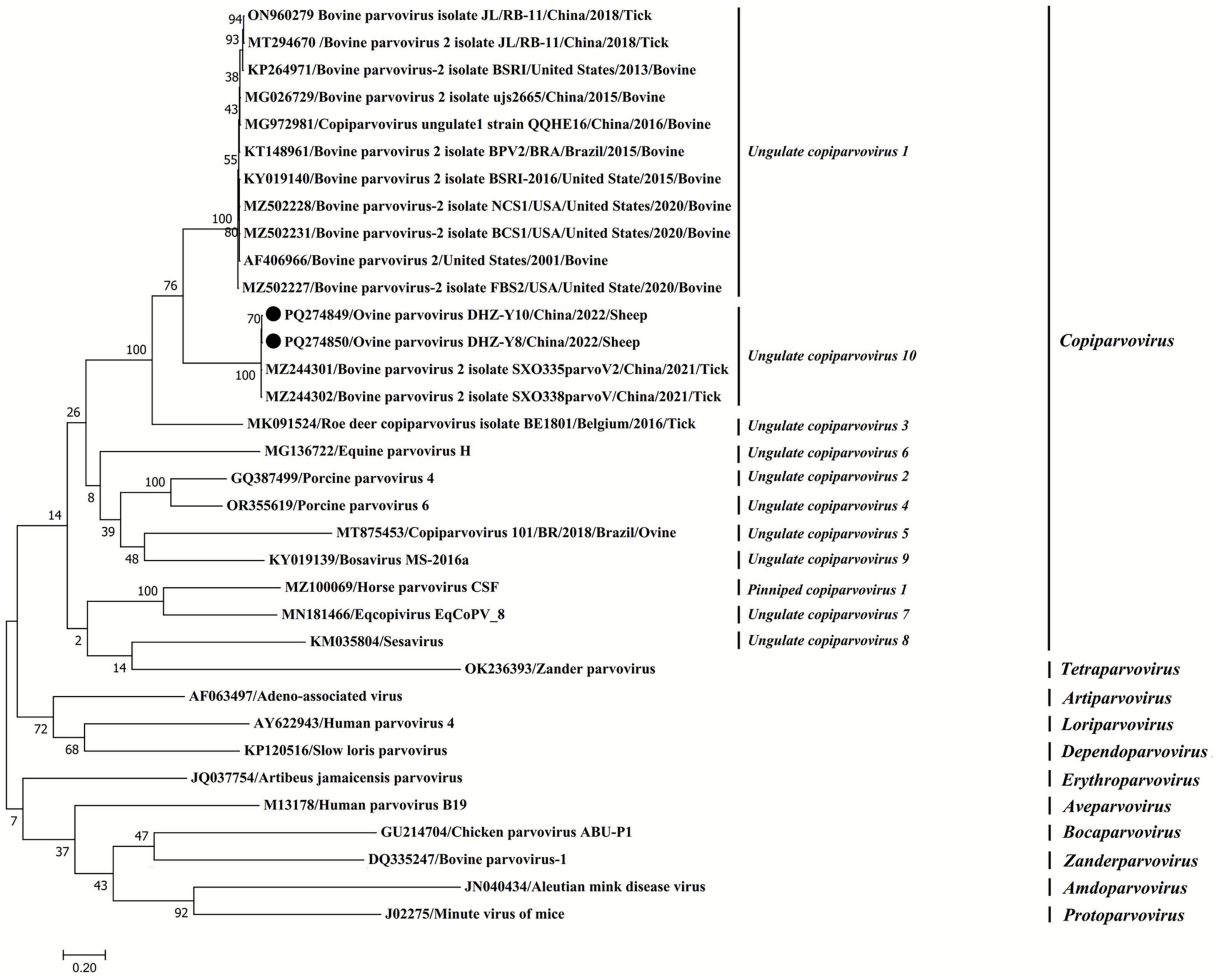
Phylogenetic analysis of the NS1 amino acid sequences of parvoviruses. Phylogenetic analysis was carried out using the maximum-likelihood (ML) method using MEGA7 software (1,000 bootstrap replicates). Fixed circles represent the viruses isolated in this study.

GenBank provided 32 reference strains of Parvovirinae subfamilies belonging to 11 genera (*Erythroparvovirus*, *Artiparvovirus*, *Tetraparvpvirus*, *Dependoparvovirus*, *Bocaparvovirus*, *Loriparvovirus*, *Amdoparvovirus, Copiparvovirus*, *Aveparvovirus*, and *Protoparvovirus*). The detailed data (GenBank accession numbers) of the sequences utilized in this study are provided in Figures 1, 2. The MegAlign software in Lasergene v7.1 was used to assess these sequences. Furthermore, the maximum-likelihood method was used to generate phylogenetic trees for all target sequences. To ensure its accuracy, the tree topology reliability was evaluated using 1,000 bootstrap replicates (16).

## Results

### Overview of the virome

**Figure 3:**
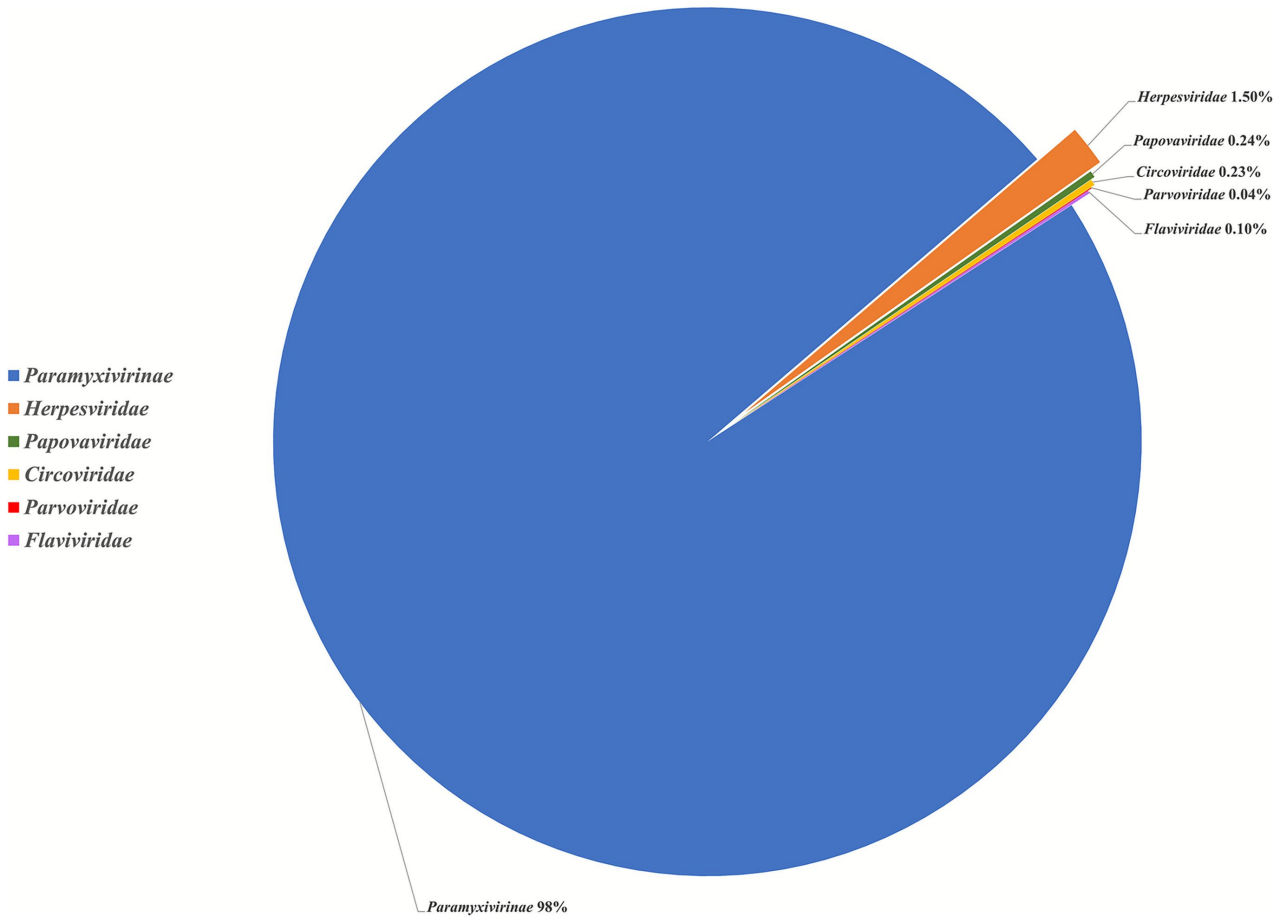
The pie chart of the read classification to different virus families.

Metagenomic analysis of 106 samples collected from Dahe town generated 230,039,218 reads. Of these, 219,170 (0.09%) reads were annotated to mammalian viruses, which were identified to be associated with six families, including *Paramyxivirinae* (peste des petits ruminants vaccine strain Clone9), *Herpesviridae* (macacine betaherpesvirus, equid gammaherpesvirus, myotis gammaherpesvirus, human alphaherpesvirus, and bovine herpesvirus), *Papillomaviridae* (papillomavirus), *Circoviridae* (circovirus), *Parvoviridae* (bovine parvovirus), and *Flaviviridae* (bovine viral diarrhea virus) (Figure 3). Furthermore, 32 reads (117 nt) shared 100% nt identity with the nonstructural protein gene of bovine parvovirus 2 (SXO335parvoV2). This is the first identification of this virus in sheep, previously known only from ticks.

### Viral detection

To assess whether the sheep and cattle were infected with the parvovirus, qPCR was performed to amplify the *NS1* gene. The results confirmed the presence of parvovirus in sheep sera from Dahe Town (CT < 22), with positivity rates of 13.78% (43/312) in 2022, 3.92% (8/204) in 2023, and 15.46% (45/291) in 2024 (Table 1). However, qPCR testing showed no evidence of viral infection in 410 samples from cattle in Dahe Town, nor in 524 sheep and 670 bovine samples from adjacent pastures in Yiwu County, Hami City, Xinjiang (CT > 35) (Table 1). Moreover, all tested samples (737 sheep and 700 cattle samples) from Tacheng City and Akesu City in Xinjiang were negative for this virus (CT > 35) (Table 1).

### Isolation of the virus and identification

To obtain data on the biological characteristics of this parvovirus, serum from virus-positive sheep was inoculated into SK and LT cells. The CPE was observed daily, and no CPE was detected following six passages. Furthermore, qPCR did not identify viral nucleic acids in cells inoculated virus-positive sheep serum.

### Phylogenetic analysis and the comparison of whole genome sequences

**Figure 4:**
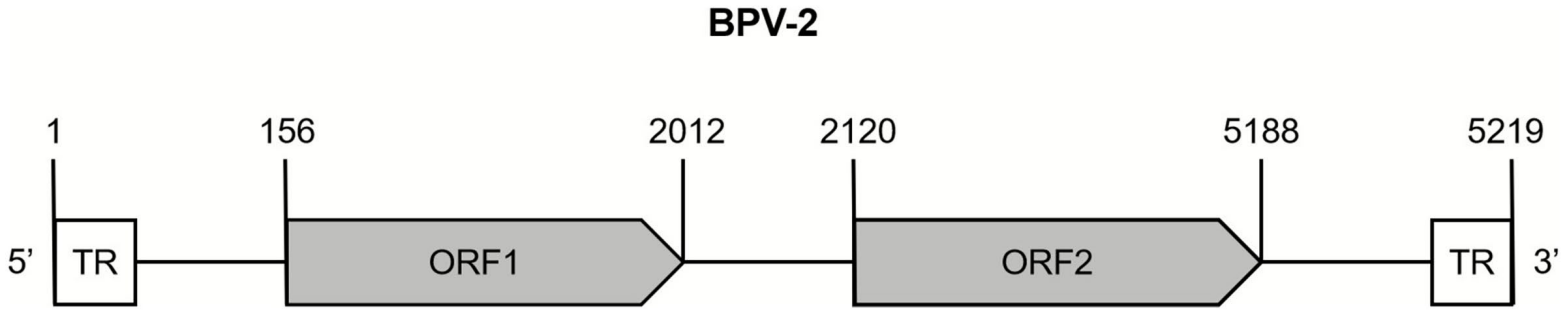
The genome structure of the novel *Ungulate copiparvovirus 10* (GenBank accession numbers PQ274849 and PQ274850) identified in sheep from China.

To assess the molecular features of OVPV, 2 whole genomes of OVPVs (DHZ-Y8 and DHZ-Y10 registered under the accession numbers PQ274849 and PQ274850) were obtained from viral-positive sera. These *OVPVs* indicated two ORFs measuring 1,857 and 3,069 nt long, respectively (Figure 4). The genome organization analysis revealed that the identified virus belonged to the subfamily Parvovirinae. Furthermore, it was found that the 1,857-nt long ORF present in the 5′ site putatively encodes NS1 protein, whereas the 3,069-nt ORF in the 3′ site encodes the putative capsid protein VP (Figure 4). Moreover, 100% nt homology was observed between the whole-genome sequences of *OVPVs*. The novel virus shared 41.6%–99.3% nt homology with 22 reference strains from the genus *Copiparvovirus* in the subfamily *Parvovirinae*. It was also observed that the 2 *OVPVs* and the 2 reference strains of bovine *parvovirus*, SXO335parvoV2, and SXO338parvoV, had a 99.3% nt similarity; however, they shared <60.9% nt identity with the other 20 reference strains from the genus *Copiparvovirus.* The phylogenetic assessment of the parvovirus’s whole genome sequence revealed that the two *OVPVs* were significantly associated with SXO335parvoV2 and SXO338parvoV (Figure 1).

### Sequence alignment of the NS1 protein

Based on the ICTV classification guidelines, parvoviruses are categorized as the same species if their NS1 proteins share >85% aa sequence similarity. NS1 proteins from the same genera must exhibit a minimum of 35%–40% aa sequence identity with >80% coverage between any two members. The sequences of the NS1 protein from two OVPVs and their representative strains of *Tetraparvovirus*, *Erythroparvovirus*, *Artiparvovirus*, *Dependoparvovirus*, *Aveparvovirus*, *Loriparvovirus*, *Amdoparvovirus, Bocaparvovirus*, *Copiparvovirus*, and *Protoparvovirus* genera were aligned via the MegAlign in Lasergene v7.1. Here, the NS1 aa sequences of two OVPVs share 99.8–99.9% homology with SXO335parvoV2 and SXO338parvoV, which remain unclassified members of the genus *Copiparvovirus*, but share 48.5%–72.8% homology with the other 10 species in the *Copiparvovirus* genus. In this study, the identified OVPV belonged to a new species within the *Copiparvovirus* genus, and was termed *Ungulate copiparvovirus 10*.

The phylogenetic assessment was carried out using the putative NS1 protein’s aa sequence and representative sequences of each genus of the Parvovirinae subfamily. The Neighbor-Joining method and the Tamura–Nei (1993) model were employed to assess the evolutionary history. The phylogenetic tree grouped 11 genera into defined clusters with high bootstrap support values (Figure 2). The NS1 aa phylogeny was used for taxonomic classification of the Parvovirinae subfamily. The OVPV’s DHZ-Y8 and DHZ-Y10 isolates, as well as the SXO335parvoV2 and SXO338parvoV strains, were clustered within a novel clade of the genus *Copiparvovirus*, indicating the highest homology with *copiparvovirus ungulate 1* (Figure 2). The phylogenetic analysis of complete genomes and NS1 aa sequences of parvovirus indicated that the OVPVs of *Ungulate copiparvovirus 10* identified in this study, along with tick-derived parvoviruses such as bovine parvovirus isolate JL/RB-11, Roe deer *copiparvovirus* isolate BE1801 of *Ungulate copiparvovirus 3,* and bovine parvovirus 2 isolate JL/RB-11 of *Ungulate copiparvovirus 1*, exhibit a relatively close genetic affinity (Figures 1, 2). These results imply that the identified OVPVs may have originated from ticks.

## Discussion

Members of the family *Parvoviridae* are nonenveloped, small ssDNA viruses with a ~5-kb genome, divided into the subfamilies *Hamaparvovirinae*, *Densovirinae*, and *Parvovirinae* (1, 2). Viruses in the subfamily of *Parvovirinae* infect vertebrate hosts and are distributed in 11 genera (1, 2). In recent years, many novel parvovirus species have been identified in different hosts, including roe deer, horses, sea lions, sheep, ticks, etc. (2–13). This is the first study to identify and report a new parvovirus, OVPV, in sheep sera from China.

Several studies have identified various parvoviruses in ticks (7). The OVPV identified in this study shared a high genetic identity with the parvovirus found in ticks, suggesting that the OVPV might have originated from ticks. However, PCR analysis identified OVPV only in sheep sera from Dahe Town, whereas bovine sera from the same area, along with sheep and bovine sera from other locations in Xinjiang Province, yielded negative results. The findings indicate that sheep in Dahe Town may act as possible natural hosts for the virus, although further research is necessary. In the future, the molecular epidemiology of OVPV will be studied in sheep and ticks to ascertain if it is mostly a sheep-associated or tick-borne virus.

This study also identified other characteristic features of parvoviruses. The predicted aa sequence of *NS1* comprised typical markers, including the replication initiator motif (I and II) and the tripartite helicase superfamily III motifs (A, B, and C), which are conserved among mammalian Densoviruses and Parvoviruses (17, 18). Furthermore, the ATP/GTP-binding site motif A (P-loop) [AG]-X4-G-K-[ST] (19, 20) was found to be well preserved in the *Ungulate copiparvovirus 10* sequences.

*Ungulate copiparvovirus 6*, *7*, and *8* were first discovered in horses with serum hepatitis with Theiler’s disease (9–13), neurological signs, and respiratory diseases. *Ungulate copiparvovirus 4* promotes porcine abortion (4, 5). *Ungulate copiparvovirus 2*, *5*, and *Pinniped copiparvovirus 1* have been detected in sick pigs, sheep, and sea lion samples, respectively, but the pathogenic potential of the three copiparvoviruses remains undetermined (4–6, 8). *Ungulate copiparvovirus 1* and *3* were identified in ticks and healthy animals (3, 7). This study isolated *Ungulate copiparvovirus 10* from healthy sheep serum samples, and its pathogenicity requires further study.

The successful isolation and cell culture of any *copiparvovirus* strain has not been reported in the previous literature (3, 6, 7). Here, it was observed that *Ungulate copiparvovirus 10* failed to propagate in SK and LT cells, underscoring the need to identify other cell lines for virus isolation and to comprehensively investigate the associated pathologies.

To our knowledge, this is the first report of parvovirus infection in sheep sera in China, analyzed *via* viral metagenomics, qPCR, and sequencing. Furthermore, this novel OVPV was classified as a new *Ungulate copiparvovirus 10* species. Genetic evolution analysis suggests that it might exist because of the habitat density of sheep and the number of ticks, which promote its transmission.

## Data Availability

The datasets presented in this study can be found in online repositories. The names of the repository/repositories and accession number(s) can be found at: https://www.ncbi.nlm.nih.gov/genbank/, PQ274849, PQ274850.

## Footnotes

1
https://talk.ictvonline.org/taxonomy/
