# Supplementary material for: Identification of a novel Ungulate copiparvovirus 10 in sheep of Hami, East Xinjiang, China
Source: Front Vet Sci. 2026 Feb 24;13:1678726. doi: 10.3389/fvets.2026.1678726 (PMC12971405; doi:10.3389/fvets.2026.1678726)
Supplement: Supplementary file 1 [file Table_1.docx]

**Table S1** Primers used in the present study

| Primer name | Amplicon bp | Primer Sequence (5'-3') | References |
| --- | --- | --- | --- |
| qPCR-OPV-F | 68 bp | GGCTTCTCAGGCTGAATCAGTAG | MZ244301 |
| qPCR-BPV-R |  | AGCCTTGAAATTGTAGCAAGTCAA |  |
| qPCR-BPV-Probe |  | FAM-ATCAGTGGAAATAAAG-MGB |  |
| PCR-BPV-F-1 | 683 bp | GCAGTTTAGGTAGGAAAAAGAGG | MZ244301 |
| PCR-BPV-R-683 |  | TATGTAAATGCCACCTGTTATGAG |  |
| PCR-BPV-F-650 | 988 bp | ACTTGGTAATACTCATAACAGGTGG |  |
| PCR-BPV-R-1638 |  | TACTGGTAATAATCATAGGAGGTGG |  |
| PCR-BPV-F-2716 | 464 bp | CCCCTACAACACCTGTCACTC |  |
| PCR-BPV-R-3180 |  | TAGCCATTGTTCTCCTTCATTTC |  |
| PCR-BPV-F-4124 | 1,347 bp | GAAGCAGGATTTATTGGTAACACAG |  |
| PCR-BPV-R-5471 |  | AACGGAACGACCAGGAAATG |  |
| PCR-BPV-F-5217 | 248 bp | AACTCCTATGTCACTTTTCAACTCC |  |
| PCR-BPV-R-5465 |  | TAGTTGGGGTTGGTGGGTG |  |
